# The Improvement of Functional State of Brain Mitochondria with Astaxanthin in Rats after Heart Failure

**DOI:** 10.3390/ijms24010031

**Published:** 2022-12-20

**Authors:** Yulia Baburina, Roman Krestinin, Dmitry Fedorov, Irina Odinokova, Ekaterina Pershina, Linda Sotnikova, Olga Krestinina

**Affiliations:** Institute of Theoretical and Experimental Biophysics, Russian Academy of Sciences, 142290 Pushchino, Moscow Region, Russia

**Keywords:** rat brain mitochondria (RBM), astaxanthin (AST), heart failure, oxidative stress, mitochondrial permeability transition pore (mPTP)

## Abstract

The relationship between neurological damage and cardiovascular disease is often observed. This type of damage is both a cause and an effect of cardiovascular disease. Mitochondria are the key organelles of the cell and are primarily subject to oxidative stress. Mitochondrial dysfunctions are involved in the etiology of various diseases. A decrease in the efficiency of the heart muscle can lead to impaired blood flow and decreased oxygen supply to the brain. Astaxanthin (AST), a marine-derived xanthophyll carotenoid, has multiple functions and its effects have been shown in both experimental and clinical studies. We investigated the effects of AST on the functional state of brain mitochondria in rats after heart failure. Isoproterenol (ISO) was used to cause heart failure. In the present study, we found that ISO impaired the functional state of rat brain mitochondria (RBM), while the administration of AST resulted in an improvement in mitochondrial efficiency. The respiratory control index (RCI) in RBM decreased with the use of ISO, while AST administration led to an increase in this parameter. Ca^2+^ retention capacity (CRC) decreased in RBM isolated from rat brain after ISO injection, and AST enhanced CRC in RBM after heart failure. The study of changes in the content of regulatory proteins such as adenine nucleotide translocase 1 and 2 (ANT1/2), voltage dependent anion channel (VDAC), and cyclophilin D (CyP-D) of mitochondrial permeability transition pore (mPTP) showed that ISO reduced their level, while AST restored the content of these proteins almost to the control value. In general, AST improves the functional state of mitochondria and can be considered as a prophylactic drug in various therapeutic approaches.

## 1. Introduction

Heart failure is a disease with serious consequences for the functions of the whole body. As a result of heart failure, brain damage can occur [1]. After heart failure, patients have been found to have both anatomical and functional brain damage. There are data showing the involvement of neurohormonal, nutritional, and inflammatory mechanisms in this complex process [2]. Mitochondrial dysfunctions are involved in the etiology of various diseases such as neurodegenerative and cardiovascular diseases, diabetes, various forms of liver and musculoskeletal diseases, sepsis, and psychiatric disorders [3]. Intracellular environmental oxidative stress, an inflammatory cascade, pH imbalances, and ionic disturbances are factors that can lead to the irreversible uncontrolled opening of mitochondrial permeability transition pore (mPTP) and, thus, can induce a mitochondria-dependent apoptotic event [4]. mPTP is a Ca^2+^-dependent channel formed by a complex of proteins that passes through the outer and inner membranes of mitochondria [5]. The structural components of the mPTP have not yet been determined; however, ANT, VDAC, and CyP-D are considered to be the regulators of mPTP [6]. Ca^2+^ plays a regulatory role in the functioning of the pore [7,8]. Moreover, the opening of the mPTP also occurs with the involvement of CyP-D, which is considered a matrix protein of mitochondria and is activated by Ca^2+^ ions. CyP-D has been thought to regulate the opening of the mPTP [9,10].

Recently, we identified the protein as a 2′,3′-cyclic nucleotide-3′-phosphodiesterase (CNPase) in rat brain mitochondria (RBM). CNPase is considered a myelin protein; however, it was found in non-myelin tissues [11]. We found that CNPase took place in the regulation of mPTP [12] and its partners are the protein regulators of mPTP (VDAC, ANT, and CyP-D) [13] and ArfGAP with dual PH domains 1 (ADAP1), also known as p42^IP4^ or centaurin α1 [14]. We hypothesized that CNPase plays the protective role in mitochondria [15].

Astaxanthin (3,3′-dihydroxy-4,4′-diketo-β-β carotene, AST) has two asymmetric carbons (3 and 3′) in its β- ionone rings, where the hydroxyl groups are attached [16,17]. The hydrophobic structure of AST consists of a conjugated polyene and terminal polar groups; therefore, AST spans across the cell membrane [18]. AST affects the biochemical processes occurring in most human organs and tissues and has antioxidant and anti-inflammatory properties [19,20]. AST easily passes through the cell membrane, reaching subcellular compartments, in particular mitochondria. It has been shown that AST added to cultured cells is transported into the mitochondria [21]. Due to its location in the cell membrane, AST protects cells from oxidative damage; it is able to scavenge free radicals; preserves the structure of the membrane; enhances the functionality of the immune system; and participates in the regulation of gene expression [22]. Wolf et al. investigated the effect of AST in PC12 cells (derived from pheochromocytoma neuroblastic cells), which are hypersensitive to oxidative stress. AST showed a protective effect against oxidative damage caused by mitochondrial dysfunction in PC12 cells [23]. Moreover, it has been proposed that AST is able to maintain and protect the integrity of the mitochondrial electron transport chain (ETC) and oxidative phosphorylation from oxidative stress [18]. Heart failure can lead to anatomical and functional damage to the brain [1]. The reason for this is often an insufficient supply of oxygen to the brain. AST has been shown to improve learning and reduce memory impairment and neuronal damage. AST increased the number of pyramidal neurons in the hippocampus and restored the normal morphology of neurons [24]. The aim of our research was to study the protective properties of AST in RBM after heart failure. Heart failure was achieved by the injection of isoproterenol (ISO) (subcutaneously) [25]. We have previously shown that isoproterenol reduced the content of proteins such as myoglobin, troponin I, lactate dehydrogenase, aspartate aminotransferase, and alanine aminotransferase in the context of cardiac dysfunction. Besides, histological analysis of the left ventricle of the rat heart showed fibrous myocardial damage in ISO-injected rats [26]. In the present study, we investigated the effect of AST on hippocampal microglia, their number and properties, the respiratory activity of mitochondria, Ca^2+^ retention capacity (CRC), mitochondrial swelling, and the change in the content of mPTP regulator proteins such as VDAC, ANT (1/2), CyP-D, CNPase, and ADAP1 in the non-synaptic brain mitochondria isolated from rat after heart failure.

## 2. Results

**Figure 1:**
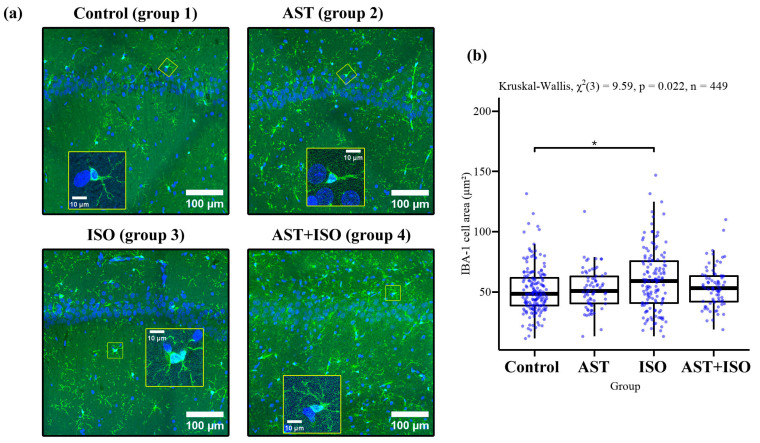
Iba-1 positive cells in the CA1 region of rat hippocampus at day three post-treatment. (**a**) Overview images of areas in CA1 region of hippocampus; (**b**) quantification of area of cell MIP in CA1 region. Statistical analysis was carried out using R v.4.0.4 and RStudio v.1.4.1106. Normality was tested using Shapiro–Wilk test. The α-value used was 0.05. Because of non-normal distribution and unequal sizes of groups in some of the comparisons, a non-parametric Kruskal–Wallis test was used with Dunn test for pairwise comparisons. The post-hoc tests were done using Bonferroni procedure.

At first, we investigated the changes in microglial cells under our experimental conditions. Figure 1a shows images of sections of hippocampus from all of the study groups after confocal microscopy. The Iba-1 staining was evaluated on the following parameters: number of cells per unit area (to account for increased number of cells), area of cell maximum intensity projection (MIP, to account for increase in cell size), and cell largest caliper (to account for appearance of rod-like microglia [27]). The number of cells per unit area was assessed by calculating the area-weighted cell densities of 4–7 regions of similar (0.15–0.25 mm^2^) size within the CA1 region. Cell shape was determined by using CellPose 2.0. Despite the fact that the number of cells per unit area did not change in all groups (data not shown), we noticed significantly larger MIP areas of cells, which indicates an increase in the size of Iba-1-positive microglial cells. Cell size increased in the ISO group rats compared to the control group, while, in the AST + ISO group, it did not differ from the control group (Figure 1b).

**Figure 2:**
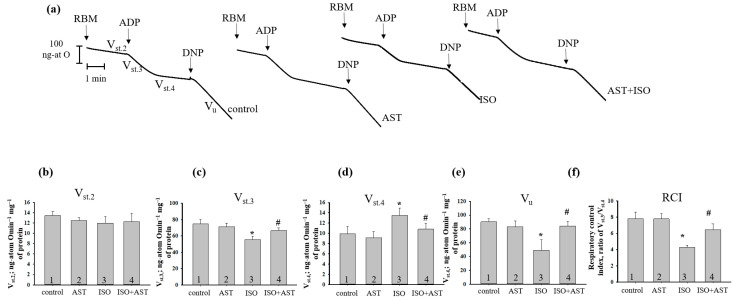
The effects of AST and ISO on respiratory activity in RBM. (**a**) Curves of respiratory activity. Arrows show the additions to RBM; (**b**–**f**) quantitative analysis of RBM respiration rate in states 2 (V_st.2_), 3 (V_st.3_), and 4 (V_st.4_). The concentration of ADP was 200 µM. DNF was 30 µM. Glutamate (5 mM) and malate (5 mM) were used as respiratory substrates. The data are presented as the means ± SDs of six independent experiments. * *p* < 0.05 significant values compared with control (group 1); # *p* < 0.05 significant values compared with RBM isolated from ISO-injected rats (group 3).

Because mitochondria are considered the first organelles to be damaged, the next step in our research was to study the respiratory activity in the rat brain mitochondria (RBM) isolated from every group (Figure 2). Figure 2a shows the curves of respiratory activities in RBM isolated from every experimental group of rats. The rates of oxygen consumption in states 2 (b), 3 (c), 4 (d), V_u_ (e), and RCI (f) were measured. According to the results, the rate of respiration in state 2 (V_st.2_) did not change in RBM isolated from either group of rats. ISO decreased the rate of oxygen consumption in state 3 (V_st.3_) by 25% compared with the value from group 1 (Figure 2c, bar 3 vs. 1). No substantial change was observed in V_st.3_ in RBM from groups 2 and 4 compared to group 1 (Figure 2c, bars 2 and 4 vs. 1). However, the V_st.3_ in RBM from group 4 increased by 16% relative to the RBM from group 3 (Figure 2b,c, bar 4 vs. 3). The oxygen consumption rate in state 4 (V_st.4_) in RBM injected with ISO accelerated by 30% compared with group 1 (Figure 2d, bar 3 vs. 1). After pretreatment with AST in combination with ISO, the oxygen consumption in state 4 (V_st.4_) in RBM did not change in comparison with control (Figure 2d, bar 4 vs. 1) and decreased by 20% relative to RBM from ISO-injected rats (Figure 2d, bar 4 vs. 3). After ISO injection, V_u_ decreased by 45% compared to the control (Figure 2e, bar 3 vs. 1). AST did not change in V_u_ relative to control (Figure 2e, bar 2 vs. 1) but increased its level by 40% in ISO-injected RBM (Figure 2e, bar 4 vs. 3). It should be noted that the RCI in mitochondria isolated from AST-treated rats (group 1) and the administration of AST with ISO injection (group 4) did not differ from the control value (Figure 2f, bars 2 and 4 vs. 1). The RCI in RBM treated with ISO decreased by 45% relative to control (Figure 2f, bar 3 vs. 1). The administration of AST with ISO injection increased the RCI by 33% compared with ISO alone (Figure 2f, bar 4 vs. 3).

**Figure 3:**
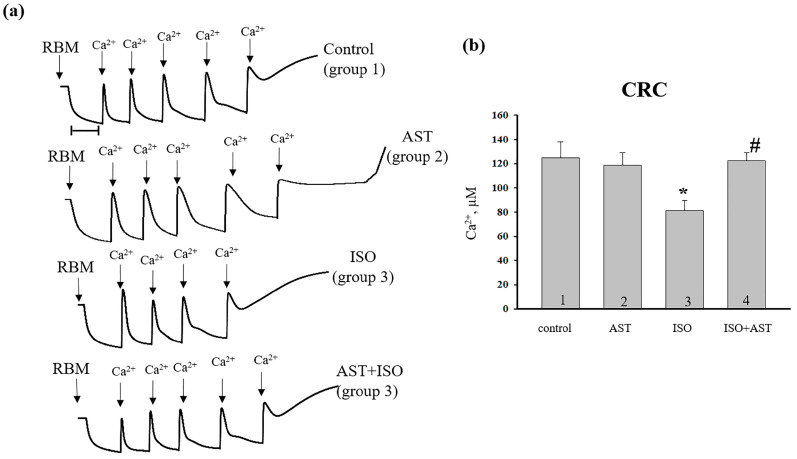
Effect of AST and ISO on the CRC in RBM upon mPTP opening. (**a**) Alterations in Ca^2+^ flows in RBM isolated from each group of rats; (**b**) quantitative analysis of the Ca^2+^ retention capacity in RBM isolated from the rats of every group. The data are presented as the means ± SDs of six independent experiments. * *p* < 0.05 indicates a significant difference in CRC relative to the control (group 1). # *p* < 0.05 shows a significant difference in CRC compared with ISO alone (group 3).

In the next step of our investigation, we examined the influence of AST on the CRC of RBM from every group of rats (Figure 3). Figure 3a shows the curves of the Ca^2+^ flows in RBM. Each addition of Ca^2+^ was 25 nmol per mg of protein. In RBM from each experimental group, the first addition of Ca^2+^ led to the active accumulation of Ca^2+^ into the mitochondria with subsequent restoration (Figure 3). In RBM from AST-treated rats and rats treated with AST along with ISO, CRC did not change relative to the control. We observed the same addition of Ca^2+^ (five pulses).

However, in RBM from ISO-treated rats, mPTP remained open after the fourth addition of Ca^2+^. Figure 3b demonstrates the quantitative changes in the CRC of Ca^2+^-loaded RBM isolated from every group of rats. We found that the CRC decreased by 30% in the RBM of ISO-injected rats (bar 3 vs. 1), while no such decrease was observed in rats treated with AST and ISO relative to control (bar 4 vs. 1). However, in RBM from rats treated with AST and ISO, the Ca^2+^ retention capacity increased by 30% compared to ISO alone (bar 4 vs. 3).

**Figure 4:**
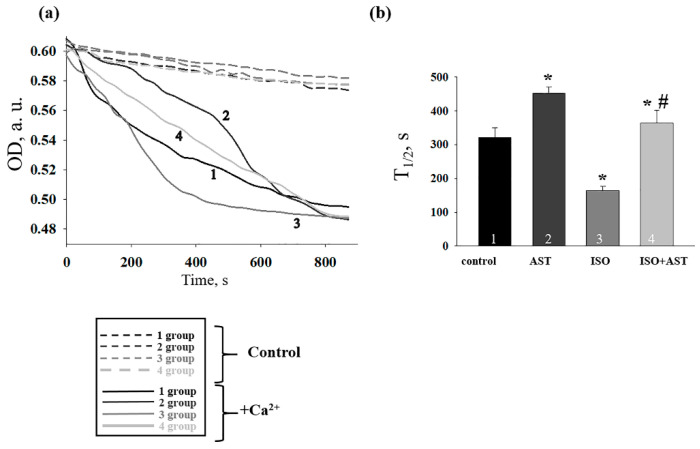
The effect of the administration of AST and ISO on the swelling of RBM. (**a**) Curves of swelling of RBM isolated from rats of each group; (**b**) the half-maximum value of mitochondrial swelling (T_1/2_). The data are presented as the means ± SDs of six independent experiments. * *p* < 0.05 indicates a significant difference in mitochondrial swelling relative to the control (group 1). # *p* < 0.05 a significant difference compared with ISO alone (group 3).

It is known that the irreversible mPTP formation is the consequence of mitochondrial swelling. Therefore, in this work, we studied the swelling of RBM isolated from every group of rats (Figure 4). Figure 4a shows the curves of Ca^2+^-activated mitochondrial swelling in RBM. The amount of Ca^2+^ was 120 mg Ca^2+^/mg mitochondrial protein. Figure 4b demonstrates the mean time to half maximum (T_1/2_) of mitochondrial Ca^2+^-activated swelling. The T_1/2_ of mitochondrial swelling in rats treated with AST increased by 50% (bar 2 vs. 1), that is, swelling was slowed down, while ISO reduced T_1/2_ by 50% compared with control (bar 3 vs. 1), i.e., the RBM swelling was accelerated. ISO injection in AST-treated rats increased T_1/2_ by 20% compared to control (bar 4 vs. 1) and 2.5-fold compared to the T_1/2_ in RBM from ISO-treated rats (bar 4 vs. 3).

Several years ago, we concluded that the protein identified as a 2′,3′-cyclic nucleotide-3′- phosphodiesterase (CNPase) is involved in the regulation of mPTP [12]. We have shown that the CNPase is co-localized with proteins such as the ADAP1 [14] and proteins regulating the functioning of the mPTP [13]. Later, we established that CNPase plays a protective role in RBM in aging [28] and in RHM after heart failure [29]. In the present study, we investigated the change in the expression of these proteins under our experimental conditions.

**Figure 5:**
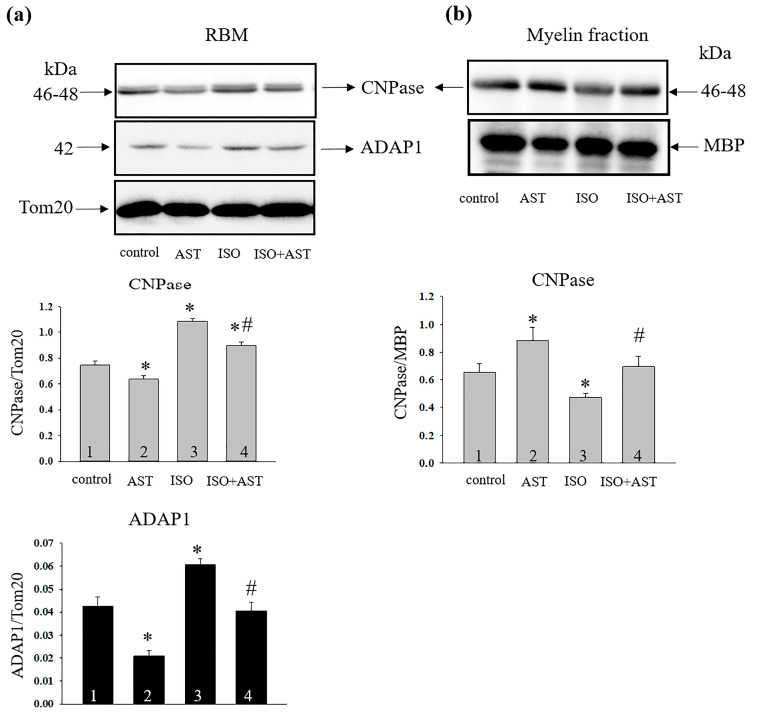
The effects of AST and ISO on the level of CNPase and ADAP1 in RBM. The immunodetection of Tom 20 and MBP was used for protein normalization. (**a**) (Upper part)—immunostaining with antibodies to CNPase, ADAP1, and Tom20; (lower part)—quantification of immunostaining by computer-assisted densitometry presents as a ratio in the level of proteins to Tom20. (**b**) (Upper part)—immunostaining with antibodies to CNPase and MBP in myelin fraction; (lower part)—quantification of immunostaining by computer-assisted densitometry presented as a ratio in the content of protein to MBP. The data are presented as the means ± SDs of five independent experiments. * *p* < 0.05 a significant difference in the protein level in comparison with the control (group 1). # *p* < 0.05 a significant difference compared to ISO alone (group 3).

Figure 5a (upper part) shows the Western blot of CNPase and ADAP1 proteins in RBM isolated from each group of rats. Antibodies to Tom20 and myelin basic protein (MBP) were used for loading control. Figure 5b (upper part) demonstrates the Western blot of CNPase in myelin fraction prepared from every group of rats. Figure 5a,b (lower parts) represents the quantitative analysis of protein bands normalized to Tom20 (for mitochondrial samples) or MBP (for myelin fraction). In RBM, the level of CNPase decreased by 14% and ADAP1 by 50% in the RBM isolated from AST-treated rats compared with the control (bar 2 vs. 1). On the contrary, ISO injection increased both CNPase and ADAP1 content by 45% and 40%, respectively, relative to control (bar 3 vs.1). The combined effect of AST and ISO was that the ADAP1 content did not change and the level of CNPase increased by 15% compared to control (bar 4 vs. 1). Under the study conditions, the levels of CNPase and ADAP1 diminished by 20% and 35%, respectively, relative to ISO alone (bar 4 vs. 3).

In myelin fraction, AST treatment increased the level of CNPase by 30% compared with control (bar 2 vs. 1), while ISO injection reduced the level of CNPase by 28% relative to the control (bar 3 vs. 1). The combined effect of ISO and AST did not change the content of CNPase compared to control (bar 4 vs. 1). However, CNPase content was enhanced by 35% compared to ISO alone (bar 4 vs. 3).

**Figure 6:**
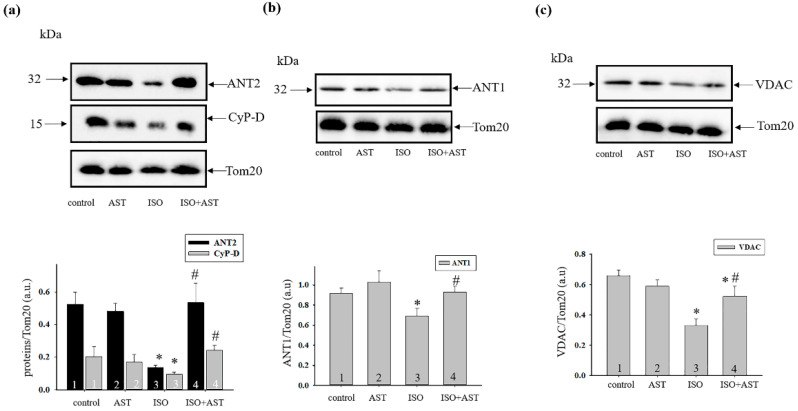
The effects of AST and ISO on the content of the mitochondrial proteins, such as adenine nucleotide translocase (ANT1 and ANT2), cyclophilin D (CyP-D), and voltage-dependent anion channel (VDAC), in RBM isolated from rats from every group. (**a**–**c**) (Upper part)—Western blots stained with corresponding antibodies. (**a**–**c**) (lower parts)—quantitative characteristic reflecting the ratio of proteins to Tom20 (loading control). The data are presented as the mean ± SDs of five independent experiments. * *p* < 0.05 indicates a significant difference in the protein level relative to the control (group 1). # *p* < 0.05 a significant difference compared to ISO alone (group 3).

An important factor in the damage of mitochondria is a change in the content of mPTP regulator proteins. Figure 6a–c (upper part) shows the Western blot of ANT1, CyP-D, ANT2, and VDAC levels in RBM. Figure 6a–c (lower parts) represents the quantitative analysis of protein bands normalized to Tom20. The administration of AST did not affect the change in the level of ANT1, ANT2, CyP-D, and VDAC compared with control (bar 2 vs. 1). The ISO injection decreased the content of ANT2, CyP-D, ANT1, and VDAC by 3.8 times, two times, 25%, and 50%, respectively, relative to control (bar 3 vs. 1). The combined effect of AST and ISO did not influence the levels of ANT1, ANT2, and CyP-D in RBM; however, the VDAC content was diminished by 20% compared with control (bar 4 vs. 1). Under these conditions (AST+ISO), the levels of ANT2, CyP-D, ANT1, and VDAC were increased 4-fold, 2.5-fold, by 36%, and by 30%, respectively, compared with ISO alone (bar 4 vs. 3).

## 3. Discussion

In this study, we investigated the effect of AST on the functional state of brain mitochondria (non-synaptic) in rats. An injection of ISO was used to induce heart failure [25]. The international scientific community has recognized this model of heart. The study of tissue lysates from the left ventricle of the rat heart showed a decrease in the content of proteins such as alanine aminotransferase, aspartate aminotransferase, lactate dehydrogenase, troponin I, and myoglobin after ISO injection (Supplementary file, Figure S1) [26]. Moreover, histological analysis of cryosections of the left ventricle of the rat heart after the injection of ISO revealed signs of fibrous transformation in the myocardium (middle zone) and, in addition, areas of fusion of swollen muscle fibers and myocardial hypertrophy (Supplementary file, Figure S2) [29]. The activation of microglia is a step-by-step process in which the cell body initially increases [30,31,32]. The appearance of amoeboid forms of microglia, characterizing activated microglia, was not observed, and rod-like microglia, while observed, were not present in large enough numbers to significantly affect the maximum caliper of the cell cohort. It is likely that the microglia involvement in response to neurological damage under our conditions was in the initial stage, which is not unexpected until day 3 after the first ISO injection.

It is known that the functional state of mitochondria declines in heart failure [33]. AST has a variety of functions and its effects have been shown in both experimental and clinical studies. AST has both lipophilic and hydrophilic properties [34]. AST reduces oxidative stress and keeps mitochondria in a more reduced state even after H_2_O_2_ stimulation. It also prevents the loss of mitochondrial membrane potential and increases the oxygen consumption in mitochondria [35].

An important characteristic of the functional state of mitochondria is respiratory activity, which indicates the effectiveness of mitochondria. It is generally accepted that a decrease in the rate of oxygen consumption in state 3 (V_st.3_) in mitochondria is associated with disruption of the electron carriers. Here, we observed that V_st.3_ decreased in RBM isolated from ISO-injected rats, however, AST increased V_st.3_ and the functional state of RBM were improved. It should be noted that ISO enhanced V_st.4_, while AST reduced this parameter. The low rate of respiration of intact mitochondria in V_st.4_ was due to the fact that a high membrane potential (created in the absence of ADP and in the presence of oxygen and substrates) prevents the transfer of protons through the inner membrane associated with the operation of the respiratory chain, thereby stopping the flow of electrons through the chain. The leakage of ions through the membrane removes the membrane potential and leads to an increase in the rate of respiration, V_st.4_. A decrease in V_st.3_ can also be a consequence of mitochondrial swelling, which leads to an increase in membrane permeability. As a result, cytochrome *c*, which is considered one of the electron carriers in the respiratory chain, is released from mitochondria [36].

The mitochondrial permeability transition pore (mPTP) across the inner and outer membranes of mitochondria is a non-specific channel for signaling or transporting various ions between the mitochondrial matrix and the cytoplasm. mPTP functions to maintain Ca^2+^ homeostasis, and it regulates oxidative stress signals and protein translocation induced by certain stimuli [4]. Therefore, we investigated the effect of AST on CRC and mitochondrial swelling in RBM isolated from rat after heart failure. We observed that the CRC decreased in RBM isolated from rats injected with ISO, whereas AST increased the CRC. In RBM isolated from rat after heart failure, the decrease in the V_st.3_ and the increase in V_st.4_ may occur due to a change in the permeability of the inner membrane of mitochondria. We noticed that ISO diminished the CRC and accelerated mitochondrial swelling, while AST abolished the effect of ISO, CRC was increased, and mitochondrial swelling was decelerated. It is generally accepted that mitochondrial swelling is closely related to the uncoupling of oxidative phosphorylation. The uncoupling of oxidative phosphorylation is accompanied by a loss of the ability of mitochondria to accumulate Ca^2+^. A decrease in the rate of respiration uncoupling in mitochondria after ISO injection was correlated with an increase in the rate of mitochondrial swelling. AST removed the effect of ISO.

It is considered that the VDAC in the outer mitochondrial membrane, the ANT in the inner mitochondrial membrane, and the CyP-D in the mitochondrial matrix are involved in the functioning of mPTP [37,38]. We predicted that the proteins such as CNPase [12] and ADAP1 (previously named p42^IP4^ or centaurin-α1) [14] participate in the regulation of mitochondrial Ca^2+^ transport and the functioning of mPTP. In addition, CNPase is co-localized with VDAC, ANT, CyP-D, and ADAP-1, and is associated with a complex respiratory chain in mitochondria [13]. Recently, we showed that the content of CNPase increased in heart mitochondria isolated from rat after heart failure. We hypothesized that CNPase has a protective function and may be the target of the AST effect in heart failure [29,39]. It was previously observed that CNPase expression in activated microglia was upregulated [40]. In the present study, it can be hypothesized that the elevated CNPase content in the mitochondria was correlated with changes in the microglia size. Earlier, we proposed that CNPase was able to protect mitochondria in aging [28]. In brain, ADAP1 is expressed in neurons, and participates in the regulation of cellular processes [41]. Moreover, ADAP1 is involved in the regulation of mitochondrial Ca^2+^ transport mechanisms and the functioning of mPTP [14]. Here, we observed that the level of CNPase and ADAP1 increased in RBM isolated from rats after heart failure, while AST abolished the effect of ISO and the protein levels decreased. In addition, we found that, during aging, the CNPase is redistributed between the myelin fraction and non-synaptic mitochondria [28]. If, in non-synaptic mitochondria during aging, the content of protein c decreased, then, in the myelin fraction, it increased. In the present study, we noticed similar dynamics. While the CNPase content in the myelin fraction decreased after ISO injection, its level increased in non-synaptic mitochondria. A study by Tan et.al. showed that CNPase protected the heart from energy starvation and proposed new therapeutic approaches to treat heart failure by influencing CNPase activity [42]. Our results on the protective role of CNPase are consistent with Tan’s data.

In mitochondria, CyP-D enhances the sensitivity of mPTP induction by causing a conformational change in the mPTP complex and increasing the affinity of the Ca^2+^ binding site of the mitochondrial matrix [43]. A decrease in CyP-D has been shown to increase mitochondrial Ca^2+^ [44]. The lack of CyP-D led to an increased susceptibility to heart failure [45]. On the other hand, VDAC is able to participate in regulation of the rate of Ca^2+^ entry into the mitochondrial intermembrane space [46], whereas ANT can prevent Ca^2+^ from entering the matrix or modulate Ca^2+^ binding to mPTP [47]. Here, we noticed that the content of VDAC, ANT (1 and 2), and CyP-D decreased in RBM from ISO-injected rats, while AST administration abolished the effect of ISO and increased the level of the protein. The changes in the level of proteins were correlated with the alteration in CRC and the swelling of mitochondria.

## 4. Materials and Methods

### 4.1. Animals and Treatments

The study used male Wistar rats, weighing 240–250 g (2 months old). The animals were kept under the same conditions. The first group was a control, the second group of rats was orally treated with AST (150 mg/kg; Natural, China) [48], and the third group of rats was injected with isoproterenol (100 mg/kg) twice in 24 h to induce heart failure [25]. The fourth group was treated with AST for two weeks, followed by two doses of ISO. All animals treated with AST and ISO were alive. AST was administered orally daily for two weeks. ISO was dissolved in physiological saline and injected twice at an interval of 24 h. The ISO was injected subcutaneously. Twenty-four animals, six rats in each group, were used in the experiment. The experiment was carried out in accordance with the regulations on conducting research on experimental animals (Order of the Ministry of Health of Russia dated 12 August 1997 No. 755). The protocol was approved by the Commission on Biological Safety and Ethics of the Institute for Theoretical and Experimental Biophysics of the Russian Academy of Sciences (March 2022, protocol N05/2022).

### 4.2. Preparation of Brain Slices for Imaging and Staining

Rats were decapitated using guillotine. The brain was extracted after decapitation and a medium segment was sectioned from one of two hemispheres. The section was then post-fixated in 4% PFA at 4 °C overnight, and then stored in PBS until slicing. Coronal slicing was done with Vibratome 1000 Series to a thickness of 100–250 μm, depending on available tissue configuration, in cold PBS.

Staining was done on whole free-floating coronal slices in 24-well plates in 1x PBS. Unspecific blocking and permeabilization was done by incubating with 10% normal horse serum (NHS, Thermo Fischer, Waltham, MA, USA) and 0.25% Triton X100 (Merck, Darmstadt, Germany) in PBS for 2 h. The carrier solution for antibodies was 5% NHS and 0.1% Triton ×100 in PBS. The antibodies used were Alexa Fluor 488-conjugated anti-tubulin Beta 3 antibody (1:500, Biolegend, San Diego, CA, USA), recombinant anti-Iba1 antibody (1:2000, Abcam, Cambridge, UK). Secondary antibody was goat anti-rabbit Alexa Fluor 594-conjugated (1:500, FineTest, FNSA-0061, Hubei, China). The primary antibodies were applied overnight at 4 °C on shaker; secondary antibodies were applied for 2 h at room temperature on shaker. This was followed by application of Hoechst 33342 (1:1000, 10 mg/mL, Thermo Fischer Scientific, Waltham, MA, USA) for 15 min and mounting in glycerin-based sealing reagent (G1401, Servicebio, Gent, Belgium) under standard coverslips. Slices were stored at 4 °C.

### 4.3. Confocal Microscopy

Slides were imaged using a laser confocal microscope (SP5, Leica Microsystems GmbH, Wetzlar, Germany) using 40× oil-immersion objective. Bit depth was 8 bits. The data were collected as a set of tiles arranged in a grid over the hippocampus, with 11% overlap at margins. Each tile was 2048 by 2048 pixels, with 2.6413 μm per pixel. There were 3 z-levels at 1 μm per level, spanning 3 μm of the slice along *z*-axis. The scanning speed was 400 Hz, pinhole size was 1 AU for each channel. The tiles were stitched in a panoramic image using Leica LAS X Mosaic Merge tool.

### 4.4. Imaging Data Analysis

The volumetric imaging data were first transformed to maximum intensity projection image (MIP) using Fiji ImageJ v.1.53q package [49]. A section of hippocampus of known area was selected in the CA1 area, if available. A number of cells were manually traced using CellPose 2.0 [50]; traces were used to train a cell-recognition model which took into account Iba-1 labeling and Hoechst staining of cell nucleus, which recognized Iba-1-positive cell bodies. The result of automatic segmentation was manually verified. The metrics were extracted using Fiji ImageJ.

### 4.5. The Isolation of Rat Brain Mitochondria

The rats were decapitated and the brains removed. Rat brain mitochondria (non-synaptic mitochondria) were isolated by method of Sims [51], modified in our laboratory [52]. Rat brain was placed in a solution containing 320 mM sucrose, 0.5 mM EDTA, 0.5 mM EGTA, 0.02% bovine serum albumin (fraction V, free of fatty acids), and 10 mM Tris-HCl, pH 7.4. The brain tissue was homogenized in a glass homogenizer. The homogenate was centrifuged at 2000× *g* for 3 min. The operation was repeated twice, and the precipitate was discarded. Brain mitochondria were centrifuged at 12,500× *g* for 10 min. The pellet was washed in a Percoll gradient (3–10–15–24%) at 31,300× *g* for 10 min. The obtained pellets of myelin fraction (upper layer) and non-synaptic mitochondria (bottom layer) were suspended in a solution containing 320 mM sucrose and 10 mM Tris-HCl, pH 7.4, and centrifuged at 11,500× *g* for 10 min for washing. The pellets were resuspended in the same buffer. All manipulations were carried out at +4 °C. Protein concentration was determined by the Bradford method (Bio Rad Protein assay; Bio-Rad, Munich, Germany) and was 30–35 mg/mL.

### 4.6. The Evaluation of Mitochondrial Functions

To identify mitochondrial functions, a multifunctional chamber (1 mL) with built-in non-selective electrodes (Ca^2+^ and Clark-type-O_2_) was used. Mitochondria (1 mg protein/mL) were incubated at 25 °C in a medium containing 125 mM KCl, 10 mM Tris (pH 7.4), and 0.4 mM K_2_HPO_4_. Glutamate (5 mM) and malate (5 mM) were used as respiratory substrates. The respiratory control index (RCI, the ratio of V_st.3_/V_st.4_) was measured in a closed chamber after the addition of 200 μM ADP. Oxygen consumption rates (V_st.2_, V_st.3_ and V_st.4_) were estimated as ng-atom O min^−1^ mg^−1^ protein. Ca^2+^ retention capacity (CRC) or threshold Ca^2+^ concentration was evaluated by the amount of Ca^2+^ pumped into mitochondria. Mitochondrial swelling was determined by change in the light scattering in the mitochondrial suspension at a wavelength of 540 nm on a Tecan I-Control Infinite 200 spectrophotometer at 25 °C. The standard incubation medium for swelling analysis contained 125 mM KCl, 10 mM Tris, 0.4 mM KH_2_PO_4_, 5 mM glutamate, and 5 mM malate. The concentration of mitochondrial protein in the well was 0.5 mg protein/mL. Swelling was initiated by adding 120 nmol Ca^2+^ per mg of protein. The swelling process was characterized by the time to reach the half-maximal light scattering signal (T_1/2_).

### 4.7. Preparation of Samples, Electrophoresis and Immunoblotting of Mitochondrial Proteins

RIPA buffer with protease inhibitors was added to brain tissue weighing 6–7 mg and brain was homogenized. The obtained samples were centrifuged at 10,000× *g* for 20 min and then the pellet was solubilized with Laemmle buffer. An aliquot of the non-synaptic mitochondria, suspension from synaptosomal fraction, and suspension from myelin fraction were solubilized with Laemmle buffer and heated at 95 °C for 3 min. The proteins from brain tissue and brain mitochondria were separated by electrophoresis (12.5% SDS-PAGE). Then, the proteins were transferred from the gel onto a nitrocellulose membrane (0.2 µm). The membrane was stained with appropriate antibodies. The monoclonal antibody to CNPase was as described previously in [53], and the monoclonal antibody to ADAP1 was as described previously in [54]. The polyclonal anti-ANT1, anti-ANT2, and anti-VDAC, as well as the monoclonal anti-CyP-D antibody, were from Abcam (Cambridge, UK). Tom20 (Cell Signaling, Danvers, MA, USA) and myelin basic protein (MBP) (Santa Cruz Biotechnology, Dallas, TX, USA) antibodies were used for normalization of proteins.

### 4.8. Statistical Analysis

For statistical analysis, the mean ± SD of at least six independent experiments was used. The statistical significance of differences between pairs of mean values was assessed using the Student–Newman–Keul test. The difference was considered significant at *p* < 0.05. Statistical analysis after confocal microscopy was carried out using R v.4.0.4 and RStudio v.1.4.1106. Normality was tested using Shapiro–Wilk test. The α-value used was 0.05. Because of non-normal distribution and unequal sizes of groups in some of the comparisons, a non-parametric Kruskal–Wallis test was used with Dunn test for pairwise comparisons. The post-hoc tests were done using Bonferroni procedure.

## 5. Conclusions

In summary, AST pretreatment improves the functional state of rat brain mitochondria after heart failure caused by ISO. ISO stimulates an increase in the size of microglia, which may imply the initial stage of brain damage. The administration of AST plus ISO injection increased the respiratory control index in rat brain mitochondria. In addition, AST enhanced the Ca^2+^ retention capacity and decreased mitochondrial swelling, which resulted in a slowdown of mPTP opening. The content of proteins (VDAC, ANT, and CyP-D) involved in the regulation of mPTP was restored. The level of CNPase decreased in RBM under the combined effect of AST and ISO, while ISO alone increased the CNPase content. The protective role of CNPase was observed. Neurological changes in the brain under the influence of AST require further research.

## Data Availability

The data presented in this study are contained within this article and online supplemental data.

## Supplementary Materials

The following supporting information can be downloaded at: https://www.mdpi.com/article/10.3390/ijms24010031/s1, Figure S1: Influence of AST and ISO on changes in the content of myoglobin, troponin I and LDH in rat heart tissue lysates. Figure S2: Histological analysis of the left ventricle of the heart of rats after AST administration and ISO injection.
