# Supplementary material for: The Improvement of Functional State of Brain Mitochondria with Astaxanthin in Rats after Heart Failure
Source: Int J Mol Sci. 2022 Dec 20;24(1):31. doi: 10.3390/ijms24010031 (PMC9820232; doi:10.3390/ijms24010031)
Supplement: Supplementary file 1 [file ijms-24-00031-s001.zip › Supplementary file.pdf]

Influence of AST and ISO on changes in the content of myoglobin, troponin I and LDH in rat heart tissue lysates.

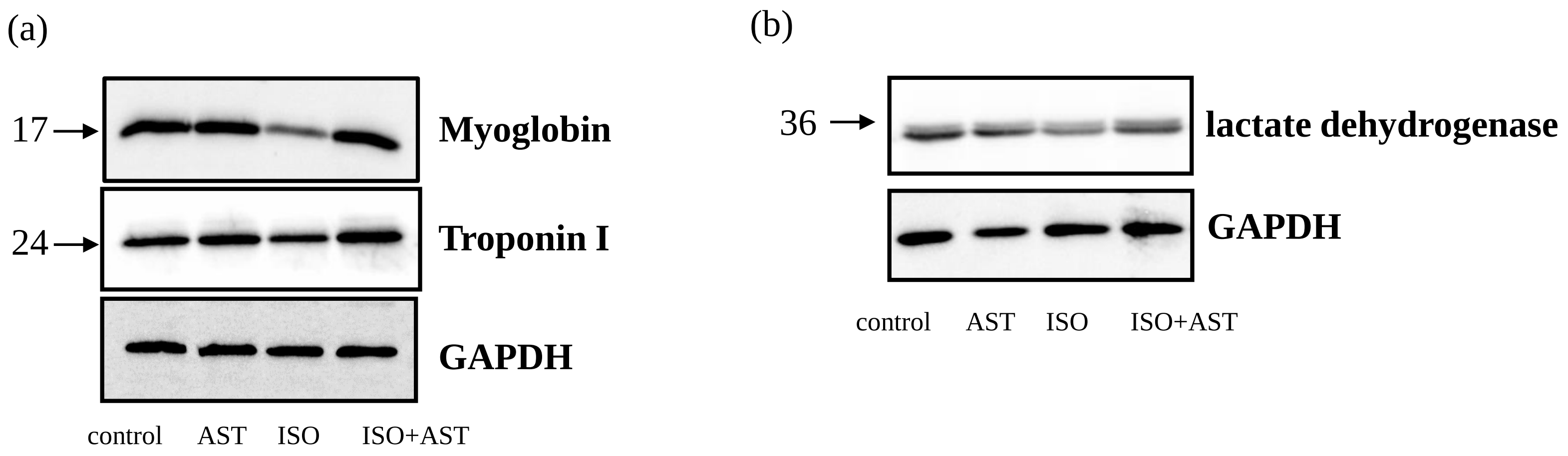

Figure S1. Protein samples were extracted and subjected to Western blot. GAPDH was used as a protein load control.
(a) and (b) - immunostaining with antibodies to myoglobin, troponin I, LDH and GAPDH;
GAPDH was used as a loading control.

Histological analysis of the left ventricle of the heart of rats after AST administration and ISO injection

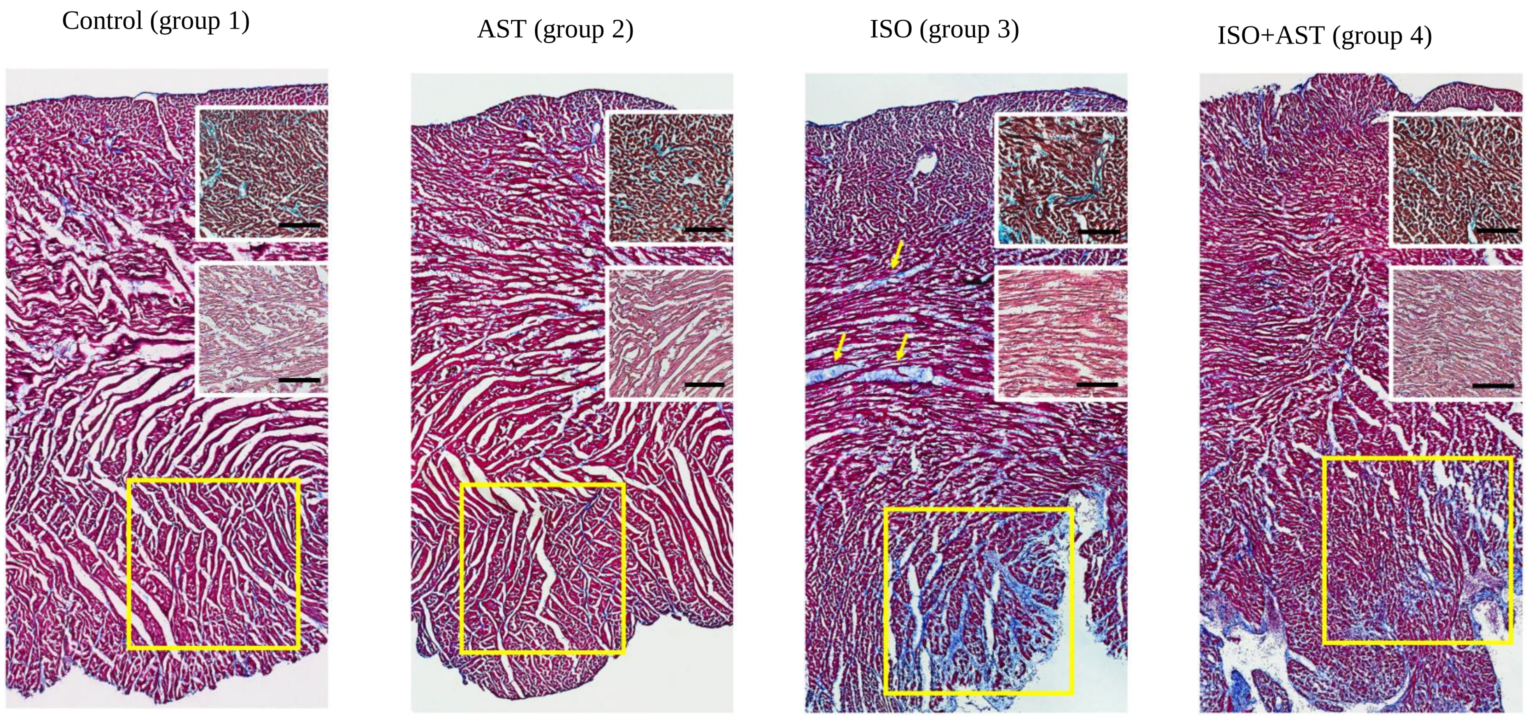

Figure S2. Light microscopy; the main images – Masson's trichrome staining (collagen/fibrosis is stained with blue, muscle and other tissues are stained with red, and cell nuclei are stained with brown);
upper insets – magnified fragments of the subendocardial zone of the myocardium with predominantly transverse section of myocardial fibers;
Lillie's trichrome staining (collagen/fibrosis is shown in blue; muscle and other tissues are in red-brown; cell nuclei are in brown-black);
middle insets – magnified fragments of the median zone of the myocardium; H&E (cell nuclei are in blue, erythrocytes are in red, muscle tissue is in pink);
lower fragments contoured in yellow are the most typical regions of the subendocardial zone of the myocardium compared;
